# Quantum Dots and Applications

**DOI:** 10.3390/ma13040897

**Published:** 2020-02-18

**Authors:** Chang-Yeol Han, Hyun-Sik Kim, Heesun Yang

**Affiliations:** Department of Materials Science and Engineering, Hongik University, Seoul 04066, Korea; honduf@naver.com

**Keywords:** quantum dots, photoluminescent, electroluminescent, photovoltaic, luminescent solar concentrator, charge transfer, photodetector

## Abstract

It is the unique size-dependent band gap of quantum dots (QDs) that makes them so special in various applications. They have attracted great interest, especially in optoelectronic fields such as light emitting diodes and photovoltaic cells, because their photoluminescent characteristics can be significantly improved via optimization of the processes by which they are synthesized. Control of their core/shell heterostructures is especially important and advantageous. However, a few challenges remain to be overcome before QD-based devices can completely replace current optoelectronic technology. This Special Issue provides detailed guides for synthesis of high-quality QDs and their applications. In terms of fabricating devices, tailoring optical properties of QDs and engineering defects in QD-related interfaces for higher performance remain important issues to be addressed.

Colloidal quantum dots (QDs), which are nano-sized semiconductor crystals, have been regarded as attractive materials in a variety of applications such as field-effect transistors [1], light-emitting diodes (LEDs) [2], and photovoltaic panels [3]. Size-dependent band gap of the QDs from quantum confinement effects is what makes them stand out among other bulk materials. This strong interrelation between the size and the band gap of the QDs was first suggested by Luis E. Brus in Bell Laboratories [4,5] only few years after the original discovery of the QDs by Alexey I. Ekimov in the early 1980s [6]. According to Brus, band gaps of bulk semiconductors could be changed when the bulk semiconductor materials were made into QDs (via a size reduction to the nanoscale via bottom-up synthesis) because of additional energy generated from spatial confinement of electron and hole pairs in the QDs. In other words, optical properties such as absorption and emission wavelength (or energy) of the QDs can be tuned extensively, typically in the range from ultraviolet to near-infrared, via a simple control of their particle size and composition. On the contrary, engineering the optical properties of bulk semiconductor materials by decreasing their particle size (from microscale to sub-microscale) is limited.

In the early stage of QD research, QDs without surface-covering shells exhibited very poor photoluminescent (PL) properties and environmental stability due to their high surface area/volume ratio compared to their bulk counterparts. Once core/shell heterostructures were introduced to the QDs in order to protect surfaces of their cores against degradable environments, their PL quantum yield (QY) and stability were greatly improved. When the interfacial stress, due to a large lattice mismatch, between the core of the heterostructured QD and its outer shell was relieved by inserting an intermediate shell, outstanding PL characteristics of ultra-high QY and narrow emission bandwidth were observed. Owing to the unique and advantageous PL characteristics of such multi-shelled QDs, their applications, especially in optoelectronic devices, have grown considerably. For example, the multi-shelled QDs have replaced bulk phosphors formerly used in LEDs. While different kinds of phosphors (in terms of their compositions) were required for the generation of different colors in conventional LEDs, current QD-LEDs could realize all colors in the visible region with the same composition (but with different particle sizes). Narrower emission bandwidth of the QDs compared to that of organic emissive materials allows QD-based displays to achieve a wider color gamut with higher color reproducibility. As a result, the multi-shelled QDs have been adopted in commercial displays as a form of color-converter. For next-generation displays, researchers have been studying electroluminescent (EL) type QD light-emitting-didoes (QLEDs) as well [2]. Photovoltaic devices such as solar cells and photodetectors, where materials with absorption abilities over a specific spectral range are required, are also well-matched applications for the QDs. Facile absorption spectral tunability, excellent light absorption coefficients, and low-cost solution-processability of the QDs have been accelerating the development of high-performance QD-based photovoltaic devices [3].

This Special Issue consists of four articles, and they cover a broad range of topics from synthesis and characterization of QDs to fabrication and study of operating mechanisms of QD devices. One of the articles is related to QD materials chemistry and QD-based EL devices [7]. Two articles then focus on QD-based photovoltaic devices [8,9], and the last article proposes a new driving mechanism for a QD device [10]. First, Kim et al. explored methods to tune PL wavelength of InP/ZnSe/ZnS QDs through Cu doping and their size control. By controlling concentration of Cu dopant in the InP cores of the multi-shelled QDs, the emission range of the Cu-doped InP QDs was engineered to have a broad spectrum from green to red regions. The size of the host QD (InP in this case) was controlled by different Zn halides used during its core synthesis. Unlike conventional methods to control the size of the QDs, such as varying QD growth time or temperature, different Zn halides utilized in the synthesis influence the surface reaction rate to determine the final size of the host QD. Moreover, the team fabricated QLEDs where the synthesized Cu-doped InP QDs were incorporated. This article can serve as a good guide on how to improve the efficiency of heavy-metal-free QDs [7]. Moving to the articles concerning QD-based photovoltaics, Zhang et al. presented a device architecture-modified phototransistor with improved performance where perovskite CsPbBr_3_ QDs-ZnO QD film hybrids were employed. The newly introduced ZnO QD film with high electron mobility, which functioned as a charge transport channel, facilitated the electron flow from the light absorption layer to the electrode. The presence of the ZnO film greatly improved the performance of the phototransistor when compared to that without the ZnO film. For instance, the on/off ratio of the device was enhanced by a factor of 560. This article demonstrates the importance of well-designed architectures for high-performance devices. [8]. Moraitis et al. evaluated the correlation between the absorption bandwidth of several state-of-the-art QDs and the transmitted sunlight spectrum by Monte Carlo simulations. They calculated the color rendering index (CRI) and the correlated color temperature of eight different QDs (CdSe/CdS, Mn^2+^-doped ZnSe/ZnS, Cu-doped CdSe, PbS/CdS, CuInSeS/ZnS, AgInS/ZnS, Silicon, and Carbon QDs) to choose suitable candidates for luminescent solar concentrators. The study shows that CuInSeS/ZnS, AgInS/ZnS, and Silicon QDs with extended absorption spectra can achieve the high CRI values required for luminescent solar concentrators. One of the challenges to be overcome will be acquiring technology to tailor the transmission spectrum of QDs [9]. Finally, Kim et al. analyzed the charge transfer mechanism at the interface of PbSe QD/ZnO, which is widely used in solar cells. They suggested that two processes of band-to-band charge tunneling and thermally activated charge transport were responsible for the observed interfacial contact properties. While the initial carrier hopping (from ZnO to PbSe) is governed by band-to-band tunneling, channel transport was dominated by thermally activated transport. This article stresses that for further device optimization defect engineering at the interface is crucial [10]. We believe this Special Issue will act as a stepping-stone for further advances in QDs and their devices in various fields.

## References

[1] Hetsch F., Zhao N., Kershaw S.V., Rogach A.L. (2013). Quantum dot field effect transistor. Mater. Today.

[2] Yang Z., Gao M., Wu W., Yang X., Sun X.W., Zhang J., Wang H.-C., Liu R.-S., Han C.-Y., Yang H. (2019). Recent advances in quantum dot-based light-emitting devices: Challenges and possible solutions. Mater. Today.

[3] Kramer I.J., Sargent E.H. (2011). Colloidal quantum dot photovoltaics: A path forward. ACS Nano.

[4] Brus L. (1984). Electron-electron and electron-hole interactions in small semiconductor crystallites: The size dependence of the lowest excited electronic state. Chem. Phys..

[5] Brus L. (1986). Electronic wave functions in semiconductor clusters: Experiment and theory. J. Phys. Chem..

[6] Ekimov A.I., Onushchenko A.A. (1981). Quantum size effect in three-dimensional microscopic semiconductor crystals. JETP Lett..

[7] Kim H.-J., Jo J.-H., Yoon S.-Y., Jo D.-Y., Kim H.-S., Park B., Yang H. (2019). Emission enhancement of Cu-doped InP quantum dots through double shelling scheme. Materials.

[8] Zhang X., Li Q., Yan S., Lei W., Chen J., Qasim K. (2019). A novel phototransistor device with dual active layers composited CsPbBr_3_ and ZnO quantum dots. Materials.

[9] Moraitis P., van Leeuwen G., van Sark W. (2019). Visual appearance of nanocrystal-based luminescent solar concentrators. Materials.

[10] Kim M., Han C.-Y., Yang H., Park B. (2019). Band to band tunneling at the zinc oxide (ZnO) and lead selenide (PbSe) quantum dot contact; interfacial charge transfer at a ZnO/PbSe/ZnO probe device. Materials.

